# Yeast protein as a fishmeal substitute: impacts on reproductive performance, immune responses, and gut microbiota in two sow hybrids

**DOI:** 10.3389/fcimb.2025.1579950

**Published:** 2025-04-22

**Authors:** Pan Zhou, Qi Liu, Yang Zhao, Yachao Wu, Jianbo Shen, Tao Duan, Long Che, Yong Zhang, Honglin Yan

**Affiliations:** ^1^ School of Life Science and Engineering, Southwest University of Science and Technology, Mianyang, Sichuan, China; ^2^ Animal Breeding and Genetics Key Laboratory of Sichuan Province, Sichuan Animal Science Academy, Chengdu, China; ^3^ College of Animal Science and Technology, Henan University of Animal Husbandry and Economy, Zhengzhou, Henan, China

**Keywords:** yeast protein, reproductive performance, immune response, gut microbiota, sow hybrid

## Abstract

**Introduction:**

The persistent African swine fever epidemic has significantly compromised China’s swine production. To accelerate production recovery, commercial farms are increasingly adopting retention of two-way backcross sows (Landrace × Yorkshire × Landrace, LLY) for breeding. This study aimed to investigate the effects of yeast protein, an emerging sustainable protein source, on reproductive performance, immune responses, and gut microbiota in two-way crossbred sows (Landrace × Yorkshire, LY) and LLY sows.

**Methods:**

The experiment employed a 2×2 factorial design evaluating two fixed factors: sow hybrid (LY vs LLY) and yeast protein supplementation (0% vs 2.6%). The four treatment groups were: LY sows without yeast protein supplementation (LY-C), LLY sows without yeast protein supplementation (LLY-C), LY sows with yeast protein supplementation (LY-YP), and LLY sows with yeast protein supplementation (LLY-YP). A total of one hundred healthy sows of 2-6 parities (50 LY sows and 50 LLY sows), were stratified by backfat thickness, body weight, and parity, then randomly allocated to the four treatment groups on day 105 of gestation, with 25 sows in each group. The experimental period lasted from day 106 of gestation to day 18 of lactation.

**Results and conclusion:**

Yeast protein supplementation showed no significant effects on most reproductive parameters of different sow hybrids, but reduced backfat loss by 30.5% during lactation (*P* < 0.05) and demonstrated a numerical reduction in mummification rate of fetuses (*P* = 0.06). Immunological assessments revealed that LLY sows exhibited 26.8% lower serum IgM concentration than LY sows (*P* < 0.05), while yeast protein supplementation significantly reduced serum IL-1β levels by 45.6% (*P* < 0.05) on day 18 of lactation. 16S rRNA gene sequencing analysis revealed comparable fecal microbial diversity across treatments (*P* > 0.05), though differences were observed in certain bacterial genera between LY and LLY sows during late gestation and lactation. Yeast protein supplementation enriched beneficial bacteria including *Ruminococcaceae_UCG-002*, *Rikenellaceae_RC9_gut_group*, and *Christensenellaceae_R_7_group*, while suppressing potentially detrimental bacteria such as *Family_XIII_AD3011_group* (*P* < 0.05). These findings demonstrate the practical feasibility of retaining LLY sows for commercial breeding. Yeast protein supplementation, as a substitute for fishmeal during late gestation and lactation, significantly reduced lactational backfat loss, moderately attenuated inflammatory response, and enhanced gut microbiome homeostasis through selective microbial enrichment in sows.

## 1 Introduction

In recent years, China’s swine industry has suffered substantial losses due to African Swine Fever (ASF) outbreaks (Liu et al., 2021). The ASF epidemic caused a significant reduction in the population of two-way crossbred sows (Landrace × Yorkshire, LY), resulting in severe shortage of breeding stock and inflated prices for breeding sows. Official data from the Ministry of Agriculture and Rural Affairs of China (MARA) revealed a 40.5% reduction in hog inventories and a 39.3% decrease in breeding sow populations between August 2018 (ASF onset) and August 2019 (Ma et al., 2021). To expedite production recovery, MARA and local governments have prioritized breeding herd restoration through policy interventions. Consequently, many farms have adopted three-way crossbred sows (Duroc × Landrace × Yorkshire, DLY) or two-way backcross sows (Landrace × Yorkshire × Landrace, LLY) as replacements for traditional LY sows. While existing research extensively compares reproductive performance between DLY and LY sows, studies evaluating LLY versus LY sows remain scarce. Current evidence suggests that although LLY sows exhibit marginally reduced heterosis compared to LY sows, their overall reproductive hybrid vigor remains substantial. Yu et al. (2020) demonstrated that LLY sows maintain genetic stability in both reproductive efficiency and growth traits, retaining sufficient heterosis for commercial breeding applications. However, further research is required to conclusively characterize performance differences between LLY and LY sows.

Additionally, China has long grappled with a chronic deficit in domestic protein feedstuffs, with excessive reliance on imported protein sources posing a significant barrier to sustainable development in both feed and livestock industries (Yin et al., 2019). This critical situation underscores the urgent need to identify alternative protein resources and develop innovative nutritional strategies to strengthen national food security. Among potential solutions, yeast, a single celled eukaryotic organism, and its derivatives, have emerged as promising candidates due to their rich composition of protein, amino acids, cell wall polysaccharides, and bioactive compounds (Shurson, 2018; Agboola et al., 2021). The diverse range of yeast-derived products, including live/dry yeast, purified cell wall components, and fermentation-derived cultures or extracts (Pang et al., 2022), has attracted significant attention from animal nutritionists seeking functional feed additives. Extensive research documents the beneficial effects of yeast products across various species, demonstrating improvements in growth performance, gut health, and immune modulation in poultry (Hofacre et al., 2024; Islam et al., 2024; Qiu et al., 2024; Maina et al., 2025), aquatic species (Jin et al., 2018; Zheng et al., 2021; Kong et al., 2025), and young pigs (Espinosa et al., 2023; Fan et al., 2024; Kim and Duarte, 2024). In sow nutrition, studies demonstrate enhanced reproductive or growth performance, immune function, and gut microbiota profiles in sows or their offspring through supplementation with various yeast products, including live yeast (Xia et al., 2022; Fu et al., 2024), yeast culture (Zhao et al., 2022; Liu et al., 2023), yeast extract (Gao et al., 2021; Tan et al., 2021; dos Santos et al., 2023), and yeast hydrolysates (Chang et al., 2024; Kim and Duarte, 2024), though some studies show limited effects (Chance et al., 2022; Le Floc′h et al., 2022). However, research of yeast products as a major protein source in swine diets remains in its infancy. Given its high digestibility and favorable essential amino acid profile (Fernandes et al., 1998), whole yeast and its derivatives present particular potential as alternative protein sources for swine nutrition, especially in regions experiencing shortage of conventional high-quality proteins like fishmeal and soybean meal. This study therefore aims to investigate the effects of yeast protein supplementation - a single-cell protein derived from hydrolyzed yeast cells - on reproductive performance, immune responses, and gut microbiota in LY and LLY sows. This study addresses two key objectives: to establish scientific foundation for optimizing LLY sow utilization in commercial swine production, and to provide essential insights for yeast protein application in both LY and LLY sows.

## 2 Materials and methods

### 2.1 Animal care

The research protocol was approved by The Animal Welfare Committee of Southwest University of Science and Technology under ethic approval number L2023021.

### 2.2 Animals and experimental design

The experiment employed a 2×2 factorial design evaluating two fixed factors: sow hybrid (LY vs LLY) and yeast protein supplementation (0% vs 2.6%). The four treatment groups were: LY sows without yeast protein supplementation (LY-C), LLY sows without yeast protein supplementation (LLY-C), LY sows with yeast protein supplementation (LY-YP), and LLY sows with yeast protein supplementation (LLY-YP). A total of one hundred healthy sows of 2-6 parities (50 LY sows and 50 LLY sows), were stratified by backfat thickness, body weight, and parity, then randomly allocated to the four treatment groups on day 105 of gestation, with 25 sows in each group. The experimental period lasted from day 106 of gestation to day 18 of lactation.

### 2.3 Diet and feeding

The experimental diets were formulated to meet the nutrient requirements for gestating and lactating sows as recommended by the NRC (2012), with detailed composition and nutritional levels presented in **Table 1**. Yeast protein supplementation was incorporated at 2.6%, a level determined by replacing 2.1% fishmeal in the control diet through iso-nitrogenous substitution, thereby constituting 7% of the total dietary protein sources. The yeast protein was produced via polysaccharide removal and subsequent protein concentration from dried yeast, primarily comprising microbial protein and yeast-derived metabolites. The analyzed crude protein and amino acid contents of the yeast protein are provided in **Table 2**.

**Table 1:** Ingredients and chemical composition of experimental diets.

Items	Control diet	YP diet	Items	Control diet	YP diet
Ingredients, %			Valine	0.11	0.11
Corn	61.675	60.365	Choline chloride	0.1	0.1
Wheat bran	5.00	5.00	Premix^1^	0.8	0.8
Soybean meal	22.00	22.50	Total	100.00	100.00
Fishmeal	2.10	–	Calculated nutrient level, %		
Yeast protein	–	2.60	DE, Mcal/kg	3.3	3.3
Calcium carbonate	1.30	1.35	CP	16.8	16.8
Dicalcium phosphate	1.05	1.25	CP from YP, %	0	7
Sodium chloride	0.50	0.50	Ca	0.9	0.9
Cottonseed oil	2.60	2.60	SID-CP	13.8	13.8
Glucose	2.50	2.50	SID-Lys	0.93	0.93
L-Lysine sulfate	0.17	0.19	SID-Met	0.27	0.27
DL-Methionine	0.035	0.05	SID-Thr	0.56	0.56
L-Threonine	0.045	0.055	SID-Trp	0.18	0.18
L-Tryptophan	0.015	0.030	SID-Val	0.75	0.75

^1^Mineral mixture supplied per kilogram of diets: Fe 120 mg; Cu 20 mg; Mn 60 mg; Zn 120 mg; Se 0.3 mg; I 0.5 mg; Carrier (Corn cob meal) 109mg. Vitamin mixture supplied per kilogram of diets: vitamin A 10000IU; vitamin D3 2000 IU; vitamin E 60 IU; vitamin K3 5.0 mg; vitamin B1 5.0 mg; vitamin B2 10.0 mg; vitamin B6 6.0 mg; vitamin B12 50 μg; nicotinic acid 40 mg; d-pantothenic acid 20 mg; folic acid 2.0 mg; biotin 0.2 mg; Carrier (Corn cob meal) 30mg.

YP, yeast protein.

**Table 2:** The analyzed contents of crude protein and various amino acids in yeast protein used in this experiment.

Item	Content, %	Item	Content, %
CP	57.10	Methionine	0.70
Aspartic Acid	5.13	Isoleucine	2.47
Threonine	2.62	Leucine	3.63
Serine	2.36	Tyrosine	1.85
Glutamic acid	9.78	Phenylalanine	2.14
Glycine	2.33	Lysine	3.81
Alanine	5.04	Histidine	1.13
Cystine	0.54	Arginine	2.33
Valine	2.82	Proline	1.72

From day 106 of gestation until parturition, sows were housed in individual crates and offered 3.0 kg/d of corresponding diets. After parturition, the feed allowance was 2 kg initially and increased by 1.0 kg/d until day 5 of lactation. From day 6 onward, all sows had free access to feed and water until weaning.

### 2.4 Measurements and sample collection

After parturition, the following parameters were recorded for each sow: number of total born piglets, number of live-born piglets, number of normal-born piglets (piglets birth weight > 800 g), and piglet birth weight. These data were used to calculate the litter birth weight, coefficient of variation (CV) of piglet birth weight, stillborn rate, mummification rate, and intrauterine growth restriction (IUGR, piglets birth weight < 800 g) rate. Piglets were weighed individually at birth, and on day 7, 14, and 18 of lactation, to calculate piglet or litter weight gain, and to estimate milk yield following the method of Hansen et al. (2012). Sow backfat thickness was measured 65 mm to the left side of the dorsal midline at the last rib (P2) using an ultrasound scanner (Renco Lean-Meater; Renco Corporation, Minneapolis, MN, USA).

On the day of farrowing, 50 mL of colostrum was collected from two to five teats of each sow. On day 18 of lactation, 0.3 mL of oxytocin was injected intravenously through the ear vein, and then 50 mL of milk was rapidly collected from two to five teats. Both colostrum and milk samples were filtered through sterile gauze and stored at -20°C until further analysis. Fasting blood samples on day 113 of gestation and day 18 of lactation were drawn by jugular vein puncture into two 5 mL tubes without anticoagulant. After 2 h of room temperature coagulation, samples were centrifuged at 2,550 × *g* at 4°C for 10 min. Serum samples were harvested and stored at -20°C until analysis. Fecal samples (2 g) were aseptically collected from six randomly selected sows per group on day 113 of gestation, day 3 of lactation, and day 18 of lactation. Samples were transported on dry ice to the laboratory and stored at -80°C pending analysis.

### 2.5 Milk and plasma sample analyses

Milk composition was analyzed for fat, protein, lactose, dry matter, and solids non-fat contents with an automatic milk quality analyzer (CombiFoss FT+, Foss, Denmark). Concentrations of immunoglobulins (IgG, IgA, IgM) and inflammatory cytokines (TNF-α, IL-10, IL-17, IL-1β) in colostrum, milk, and serum were determined with commercial ELISA kits (Nanjing Jiancheng Bio-Engineering Institute, China).

### 2.6 Fecal microbial analysis

Microbial DNA was extracted from thawed stool samples using the EZNA. ^®^Stool DNA Kit (D4015, Omega, Inc., Norwalk, CN, USA) following the manufacturer’s protocol. The genomic DNA was measured for purity and integrity before sequencing. The V4 hypervariable region of the 16S rRNA gene was amplified using 515F and 806R primers according to Zhou et al. (2023). The 16S RNA gene sequencing was performed on PacBio Sequel II platform. Sequences with ≥97% similarity were clustered to the same operational taxonomic unit (OTU) using USEARCH (v10.0). Representative sequences for each OTU were selected. The Naive Bayes classifier in QIIME2 (v2020.6) was used for taxonomic classification. The relative abundance of each OTU was examined at different taxonomic levels. Alpha diversity, as well as taxonomic community assessments, were performed by QIIME2 (v2020.6). Beta diversity was analyzed by principal coordinate analysis (PCoA) to assess the diversity in samples using QIIME (v1.9.1) (Mou et al., 2025).

### 2.7 Statistics

The statistical analysis was performed using the MIXED procedure of SAS software (Version 9.3; SAS Institute Inc., Cary, NC, USA), except for stillborn rate, mummification rate, IUGR rate, and piglet preweaning mortality, where odds ratios of these traits were analyzed using the GENMOD procedure of SAS. The fixed effects in the mixed model include sow hybrid (LY vs LLY), yeast protein supplementation (0% vs 2.6%), and their interaction. Mean values were presented as least square mean ± largest SEM, except for stillborn rate, mummification rate, IUGR rate, and piglet preweaning mortality which were reported as mean and their 95% confidence limits. All variables were considered significant when *P* < 0.05, whereas 0.05 < *P* < 0.1 was considered a tendency.

For the 16S rRNA sequencing data, differences in the alpha diversity indexes between groups were analyzed by *t*-test. The permutational multivariate analysis of variance (PERMANOVA) was used on the Bray-Curtis distance matrices to assess the beta diversity between groups. The Wilcoxon rank-sum test was used to compare data of relative abundance at different taxonomic levels between groups.

## 3 Results

### 3.1 Sow and litter performance

Reproductive performance analysis (**Table 3**) revealed neither main effects of sow hybrid nor yeast protein supplementation, nor their interactive effects on majority of reproductive parameters (*P* > 0.05). Notably, LLY sows exhibited significantly lower CV of piglet birth weight compared to LY sows (19.37% vs. 22.88%; *P* < 0.01). Yeast protein supplementation demonstrated a numerical reduction in mummification rate of fetuses (0% vs. 0.87%; *P* = 0.06).

**Table 3:** The effect of yeast protein supplementation on the farrowing performance of two sow hybrids.

Items	Sow hybrid (H)		YP supplementation (YP)		SEM	*P-*value		
	LY	LLY	Control	YP		H	YP	H×YP
Total born piglets	14.22	13.46	13.82	13.86	0.62	0.18	0.95	0.83
Live-born piglets	13.28	12.91	12.98	13.22	0.56	0.47	0.64	0.54
Normal-born piglets^1^	12.31	12.32	12.17	12.45	0.51	0.98	0.55	0.12
Litter birth weight, kg	18.83	18.19	18.45	18.57	0.76	0.36	0.87	0.15
Piglet birth weight, kg	1.33	1.41	1.37	1.36	0.05	0.11	0.86	0.06
CV of piglet birth weight, %	22.88^a^	19.37^b^	20.19	22.06	1.41	< 0.01	0.15	0.40
Stillborn rate^2^, %	4.08	3.29	3.92	3.42		0.41	0.54	0.36
	[2.75;6.05]	[2.07;5.22]	[2.56;5.99]	[2.21;5.29]				
Mummification rate^3^, %	0.56	0	0.87	0		0.37	0.06	0.33
	[0.19;1.67]	[0;0]	[0.37;2.05]	[0;0]				
IUGR rate^4^, %	5.82	3.81	5.12	4.32		0.08	0.30	0.18
	[4.18;8.08]	[2.47:5.87]	[3.49;7.52]	[2.94:6.36]				

^1^piglets birth weight > 800 g.

^234^Data were binomially distributed, and hence confidence limits were given in brackets instead of SEM values.

Within a row and within a main effect, values with different letters are significantly different (*P* < 0.05).

YP, yeast protein; CV, coefficient of variation; IUGR, intrauterine growth restriction, piglets birth weight < 800 g.

Lactation performance parameters (**Table 4**) remained unaffected by either sow hybrid or yeast protein supplementation in most measured indices (*P* > 0.05). However, LLY sows demonstrated a numerical reduction in average daily feed intake (ADFI) (4.91 vs. 5.22 kg/d; *P* = 0.06). And yeast protein supplementation reduced sow backfat loss by 30.5% during lactation (0.98 vs. 1.41 mm; *P* < 0.05).

**Table 4:** The effect of yeast protein supplementation on the lactational performance of two sow hybrids.

Items	Sow hybrid (H)		YP supplementation (YP)		SEM	*P*-value		
	LY	LLY	Control	YP		H	YP	H×YP
Litter size after cross-fostering	12.01	11.71	11.85	11.88	0.22	0.27	0.93	0.10
Piglet weight, kg								
After cross-fostering	1.40	1.47	1.44	1.43	0.04	0.15	0.90	0.16
Day 7	2.55	2.66	2.63	2.58	0.06	0.15	0.56	0.34
Day 14	4.32	4.46	4.43	4.35	0.09	0.22	0.53	0.46
Day 18	5.33	5.44	5.39	5.38	0.12	0.33	0.95	0.49
Average daily gain, g	210.0	216.4	212.2	214.2	5.8	0.24	0.71	0.43
Litter weight, kg								
After cross-fostering	16.88	17.29	17.22	16.95	0.48	0.53	0.68	0.30
Day 7	30.81	31.21	31.52	30.50	0.77	0.71	0.34	0.09
Day 14	48.09	49.39	49.24	48.24	1.26	0.45	0.56	0.14
Day 18	58.02	59.44	59.23	58.24	2.23	0.50	0.64	0.22
Total weight gain	43.14	43.76	43.75	43.15	1.71	0.71	0.72	0.61
Sow backfat thickness, mm								
At parturition	15.77	15.23	15.88	15.11	0.68	0.41	0.23	0.10
At weaning	14.40	13.69	14.25	13.84	0.63	0.24	0.50	0.37
Backfat loss	1.14	1.24	1.41^a^	0.98^b^	0.21	0.61	0.03	1.00
Piglets preweaning mortality^1^, %	11.9	9.1	12.8	8.4		0.41	0.11	0.75
	[8.4;16.9]	[6.3:13.0]	[8.7;18.8]	[6.1:11.6]				
Sow ADFI, kg/d	5.22	4.91	5.06	5.08	0.17	0.06	0.89	0.82

^1^Data were binomially distributed, and hence confidence limits were given in brackets instead of SEM values.

Within a row and within a main effect, values with different letters are significantly different (*P* < 0.05).

YP, yeast protein; ADFI, average daily feed intake.

### 3.2 Milk yield and milk composition

Milk composition analysis (**Table 5**) demonstrated that neither sow hybrid nor yeast protein supplementation exerted significant influence on milk yield or majority of compositional parameters in both colostrum and milk (*P* > 0.05). Notably, LLY sows exhibited elevated milk fat content compared to LY sows in colostrum (5.78% vs 4.25%) and milk (6.77% vs 5.78%) (*P* < 0.05). Furthermore, LLY sows showed higher milk dry matter (20.98% vs 19.42%; *P* < 0.01) and solids non-fat content (14.51% vs 14.12%; *P* = 0.05) compared to LY sows.

**Table 5:** The effect of yeast protein supplementation on the milk composition and milk yield of two sow hybrids.

Items	Sow hybrid (H)		YP supplementation (YP)		SEM	*P*-value		
	LY	LLY	Control	YP		H	YP	H×YP
Colostrum, %								
Milk fat	4.25^b^	5.78^a^	5.54	4.50	0.71	0.01	0.08	0.82
Milk protein	17.49	18.88	17.66	18.71	1.40	0.22	0.35	0.74
Milk Lactose	2.73	2.54	2.73	2.54	0.26	0.34	0.38	0.76
Milk DM	28.17	31.12	29.66	29.63	1.85	0.06	0.99	0.78
Solids non-fat	23.73	25.01	23.87	24.88	1.31	0.23	0.34	0.79
Milk, %								
Milk fat	5.78^b^	6.77^a^	6.15	6.40	0.49	0.02	0.52	0.36
Milk protein	4.71	4.96	4.92	4.75	0.22	0.16	0.32	0.11
Milk Lactose	6.23	6.34	6.28	6.29	0.10	0.23	0.82	0.21
Milk DM	19.42^b^	20.98^a^	19.98	20.42	0.50	< 0.01	0.27	0.32
Solids non-fat	14.12^b^	14.51^a^	14.31	14.32	0.24	0.05	0.94	0.46
Milk yield, kg/d	9.88	10.20	9.98	9.57	0.27	0.86	0.32	0.17

Within a row and within a main effect, values with different letters are significantly different (*P* < 0.05).

YP, yeast protein.

### 3.3 Concentration of immunoglobulins and inflammatory cytokines in serum

Serum immunological profiling (**Table 6**) revealed no main effects of sow hybrid nor yeast protein supplementation on serum immunoglobulins or inflammatory cytokines on day 113 of gestation (*P* > 0.05). However, on day 18 of lactation, LLY sows exhibited 26.8% lower serum IgM concentration than LY sows (2.18 vs. 2.98 mg/mL; *P* < 0.05), and yeast protein supplementation decreased the serum IgG by 22.6% (2.43 vs. 3.14 mg/mL; *P* < 0.05) and IL-1β by 45.6% (70.40 vs. 129.53 pg/mL; *P* < 0.05) in sows. In addition, a significant interactive effect was observed between the main effects on the serum IL-10 content on day 18 of lactation (*P* = 0.02).

**Table 6:** The effect of yeast protein supplementation on concentrations of immunoglobulins and inflammatory cytokines in sow serum.

Items	Sow hybrid (H)		YP supplementation (YP)		SEM	*P*-value		
	LY	LLY	Control	YP		H	YP	H×YP
Day 113 of gestation								
IgG, mg/mL	4.04	4.29	4.35	3.97	0.79	0.69	0.53	0.40
IgA, μg/mL	155.4	131.34	153.14	133.6	27.4	0.27	0.37	0.84
IgM, mg/mL	2.40	2.69	2.59	2.49	0.55	0.51	0.83	0.78
TNF-α, pg/mL	21.02	19.88	18.14	22.76	3.00	0.63	0.06	0.12
IL-10, pg/mL	24.10	27.71	28.65	23.16	5.48	0.40	0.21	0.20
IL-17, pg/mL	5.91	6.12	5.82	6.21	0.84	0.76	0.54	0.50
IL-1β, pg/mL	51.26	61.17	63.24	49.19	13.64	0.36	0.19	0.59
Day 18 of lactation								
IgG, mg/mL	2.66	2.92	3.14^a^	2.43^b^	0.37	0.41	0.04	0.18
IgA, μg/mL	125.9	139.6	140.6	124.9	18.6	0.39	0.33	0.73
IgM, mg/mL	2.98^a^	2.18^b^	2.51	2.65	0.33	< 0.01	0.62	0.64
TNF-α, pg/mL	40.92	33.29	38.09	36.13	7.20	0.22	0.75	0.59
IL-10, pg/mL	24.45	28.05	29.29	23.21	3.70	0.26	0.06	0.02
IL-17, pg/mL	8.26	8.35	9.08	7.52	1.51	0.94	0.23	0.43
IL-1β, pg/mL	93.18	112.75	129.53^a^	76.40^b^	27	0.35	0.02	0.45

Within a row and within a main effect, values with different letters are significantly different (*P* < 0.05).

YP, yeast protein.

### 3.4 Concentration of immunoglobulins and inflammatory cytokines in colostrum and milk

Colostrum and milk immunological profiling (**Table 7**) demonstrated neither sow hybrid nor yeast protein supplementation significantly influenced majority of immunoglobulins and inflammatory cytokines in both colostrum and milk (*P* > 0.05). However, yeast protein supplementation reduced the IgG concentration in colostrum by 34.3% (2.90 vs. 4.42 mg/mL; *P* < 0.01).

**Table 7:** The effect of yeast protein supplementation on concentrations of immunoglobulins and inflammatory cytokines in sow milk.

Irems	Sow hybrid (H)		YP supplementation (YP)		SEM	*P*-value		
	LY	LLY	Control	YP		H	YP	H×YP
Colostrum								
IgG, mg/mL	3.65	3.68	4.42^a^	2.90^b^	0.59	0.95	< 0.01	0.52
IgA, μg/mL	137.0	137.1	136.2	137.8	22.1	1.00	0.93	0.77
IgM, mg/mL	3.12	3.19	2.86	3.44	0.50	0.86	0.19	0.93
TNF-α, pg/mL	17.12	17.57	16.59	18.10	3.51	0.88	0.62	0.74
IL-10, pg/mL	23.63	23.74	24.10	23.27	2.52	0.96	0.71	0.47
IL-17, pg/mL	5.74	5.61	5.78	5.56	0.92	0.87	0.78	0.78
IL-1β, pg/mL	64.96	59.51	66.71	57.76	14.93	0.67	0.49	0.21
Milk								
IgG, mg/mL	3.50	3.63	3.66	3.46	0.61	0.80	0.70	0.14
IgA, μg/mL	161.5	150.3	165.8	146.1	17.2	0.47	0.21	0.89
IgM, mg/mL	3.16	3.40	3.30	3.26	0.44	0.52	0.93	0.81
TNF-α, pg/mL	18.14	22.19	19.65	20.68	3.67	0.20	0.74	0.72
IL-10, pg/mL	18.00	21.77	19.27	20.49	2.86	0.13	0.62	0.25
IL-17, pg/mL	5.21	5.37	4.73	5.85	0.81	0.82	0.11	0.27
IL-1β, pg/mL	56.47	70.46	57.32	69.61	13.61	0.24	0.30	0.75

Within a row and within a main effect, values with different letters are significantly different (*P* < 0.05).

YP, yeast protein.

### 3.5 Analysis of the differences in fecal microbiota between two sow hybrids

**Figure 1:**
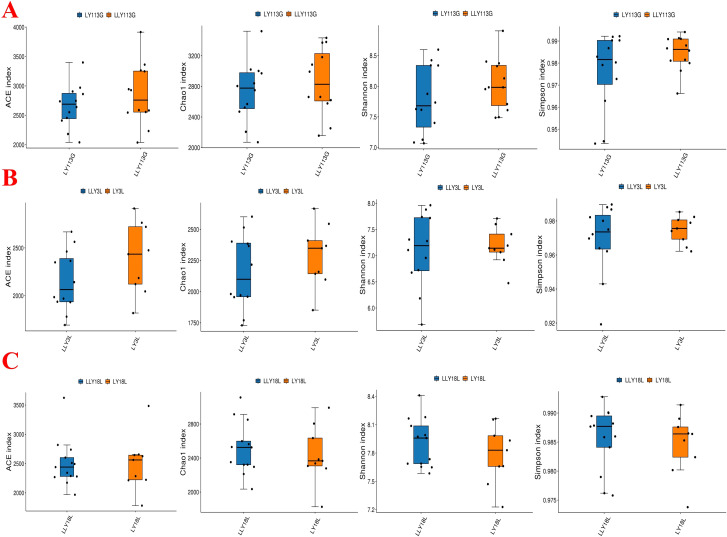
Alpha diversity index of fecal microbiota in two sow hybrids at different stages. **(A)** 113G, day 113 of gestation. **(B)** 3L, day 3 of lactation. **(C)** 18L, day 18 of lactation. LY, Landrace × Yorkshire; LLY, Landrace × Yorkshire × Landrace.

**Figure 2:**
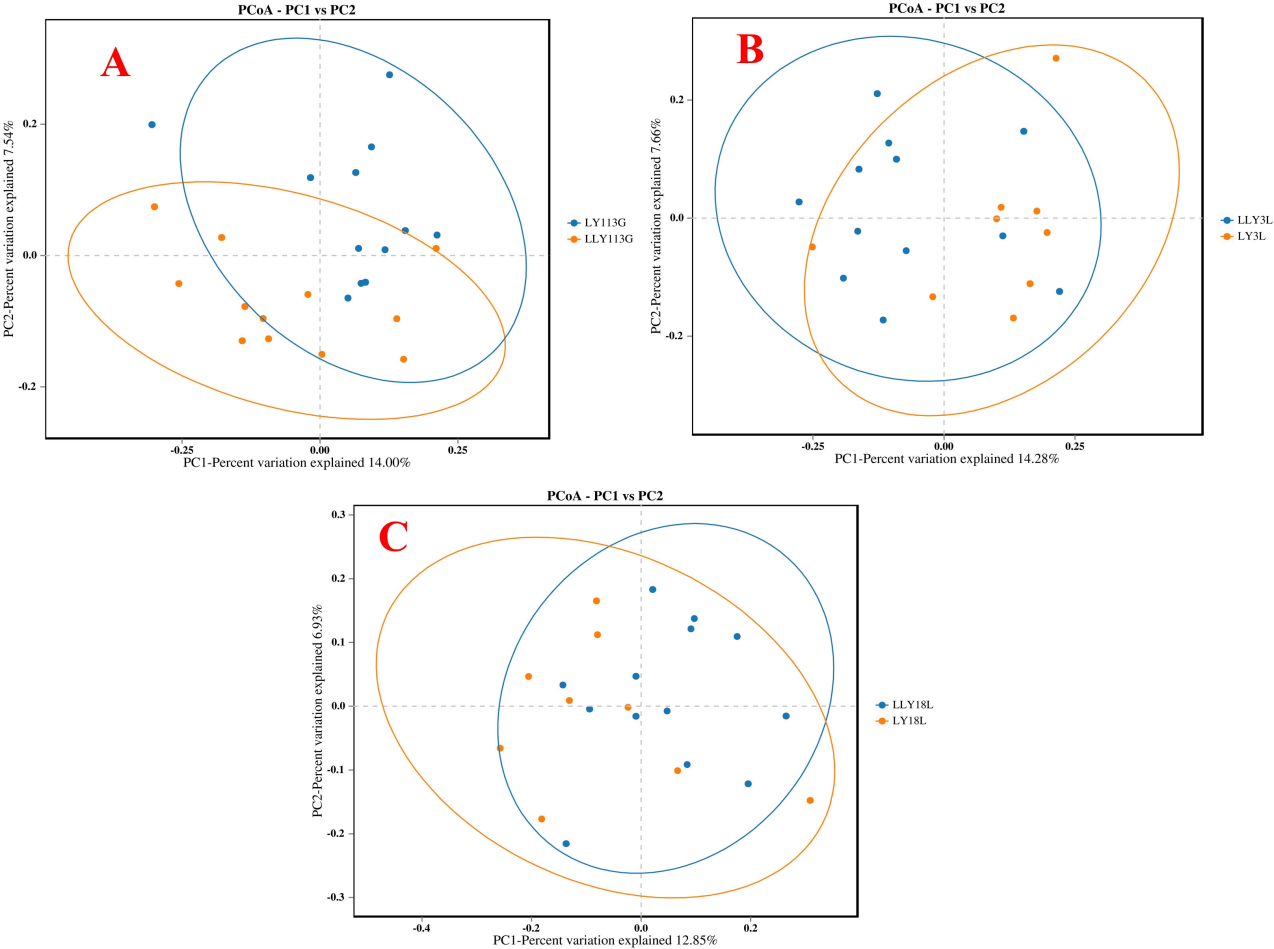
The Principal Coordinate Analysis (PCoA) of bacterial communities in two sow hybrids. **(A)** 113G, day 113 of gestation. **(B)** 3L, day 3 of lactation. **(C)** 18L, day 18 of lactation. LY, Landrace × Yorkshire; LLY, Landrace × Yorkshire × Landra.

The alpha diversity analysis revealed no significant differences in the ACE index, Chao1 index, Shannon index, and Simpson index between LY and LLY sows on day 113 of gestation, day 3 of lactation, and day 18 of lactation (**Figures 1A–C**; *P* > 0.05). Beta-diversity assessment through PCoA based on the Bray-Curtis distance matrices demonstrated no clear clustering between the two different sow hybrids at any sampling timepoint (*P* > 0.05; **Figures 2A–C**).

**Figure 3:**
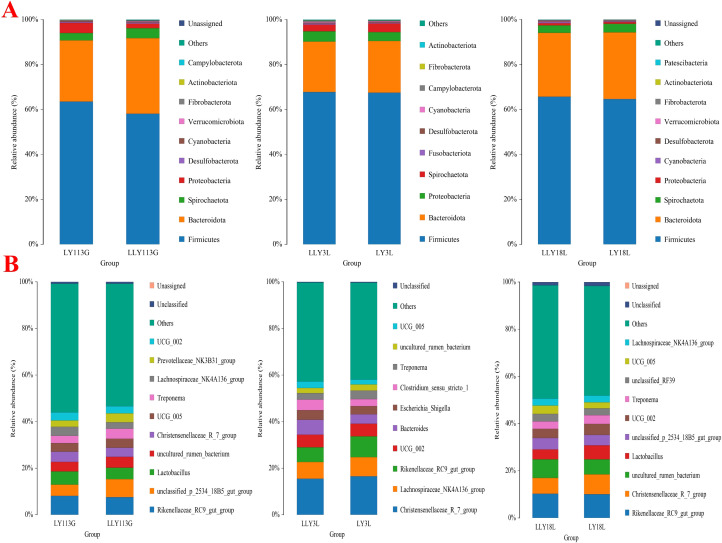
Relative abundances of top 10 bacteria at levels of phyla **(A)** and genera **(B)** in two sow hybrids at different stages. LY, Landrace × Yorkshire; LLY, Landrace × Yorkshire × Landrace; 113G, day 113 of gestation; 3L, day 3 of lactation; 18L, day 18 of lactation.

Community composition at the phylum level showed Firmicutes as the primary dominant phylum and Bacteroidetes as the secondary dominant phylum across all samples (**Figure 3A**). The bacterial community composition of the top 10 genera is displayed in **Figure 3B**. In the two sow hybrids, *Rikenellaceae_RC9_gut_group*, *unclassified_p_2534_18B5_gut_group*, and *Lactobacillus* were the top three prevalent genera on day 113 of gestation. These dominant genera shifted to *Christensenellaceae_R_7_group*, *Lachnospiraceae_NK4A136_group*, and *Rikenellaceae_RC9_gut_group* on day 3 of lactation, and further changed to *Rikenellaceae_RC9_gut_group*, *Christensenellaceae_R_7_group*, and *uncultured_rumen_bacterium* on day 18 of lactation.

**Figure 4:**
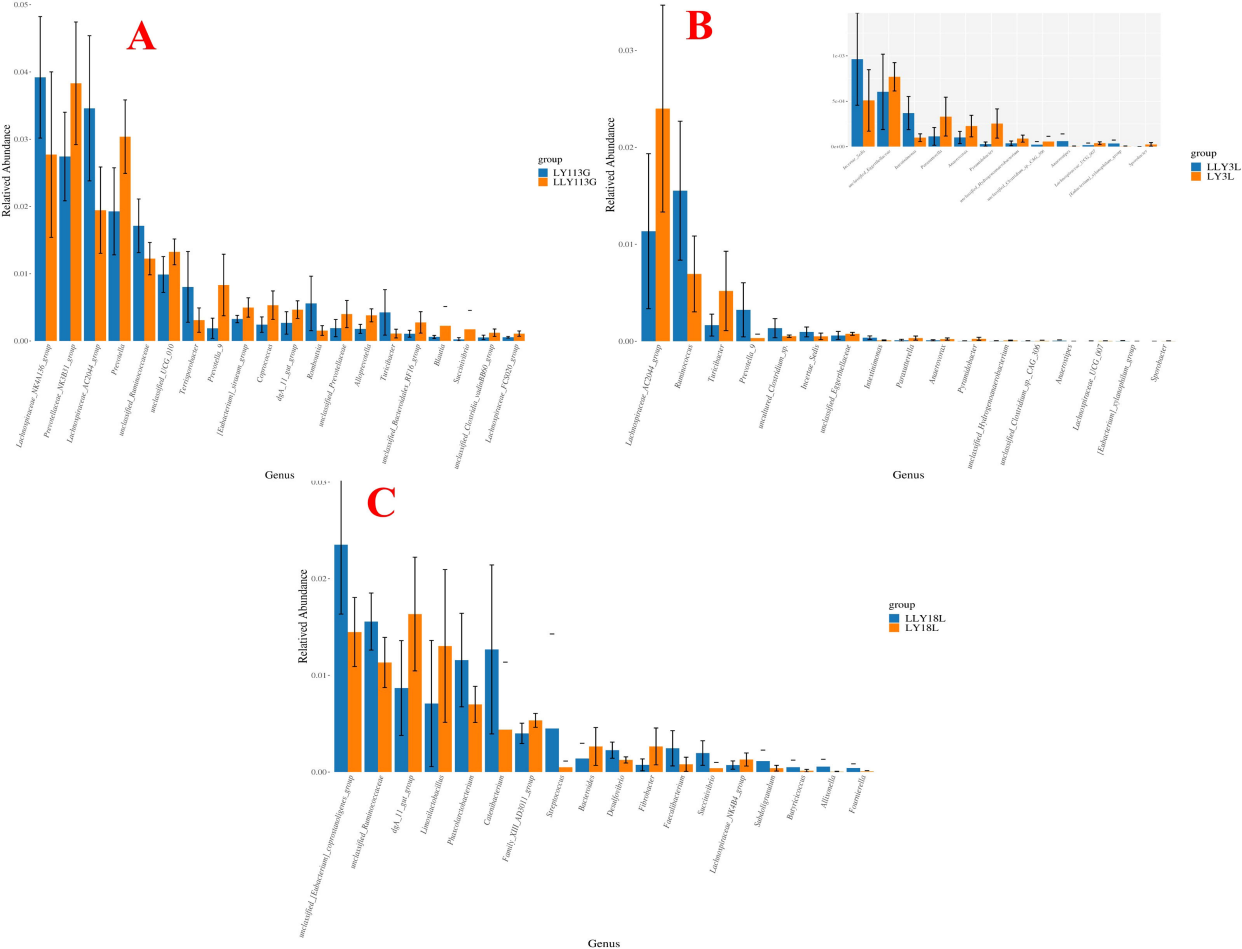
Analysis of differential bacterial genera in fecal microbiota of two sow hybrids at different stages. **(A)** 113G, day 113 of gestation. **(B)** 3L, day 3 of lactation. **(C)** 18L, day 18 of lactation. LY, Landrace × Yorkshire; LLY, Landrace × Yorkshire × Landrace.

Wilcoxon rank-sum test for the differential microbial genera in the feces of different sow hybrids are shown in **Figure 4**. On day 113 of gestation, LLY sows exhibited reduced relative abundances of *Lachnospiraceae_NK4A136_group*, *Lachnospiraceae_AC2044_group*, and *unclassified_Ruminococcaceae*, contrasting with elevated abundances of *Prevotellaceae_NK3B31_group*, *Prevotella*, and *unclassified_UCG_010* compared to LY sows (*P* < 0.05; **Figure 4A**). On day 3 of lactation, LLY sows demonstrated enriched relative abundances of *Ruminococcus* but depleted *Lachnospiraceae_AC2044_group* compared to LY sows (*P* < 0.05; **Figure 4B**). On day 18 of lactation, LLY sows showed significantly higher abundances of *unclassified_[Eubacterium]_coprostanoligenes_group*, *unclassified_Ruminococcaceae*, *Phascolarctobacterium*, and *Catenibacterium*, alongside reduced *dgA_11_gut_group* and *Limosilactobacillus* abundances compared to LY sows (*P* < 0.05; **Figure 4C**).

### 3.6 Effects of yeast protein supplementation on the fecal microbiota of sows

**Figure 5:**
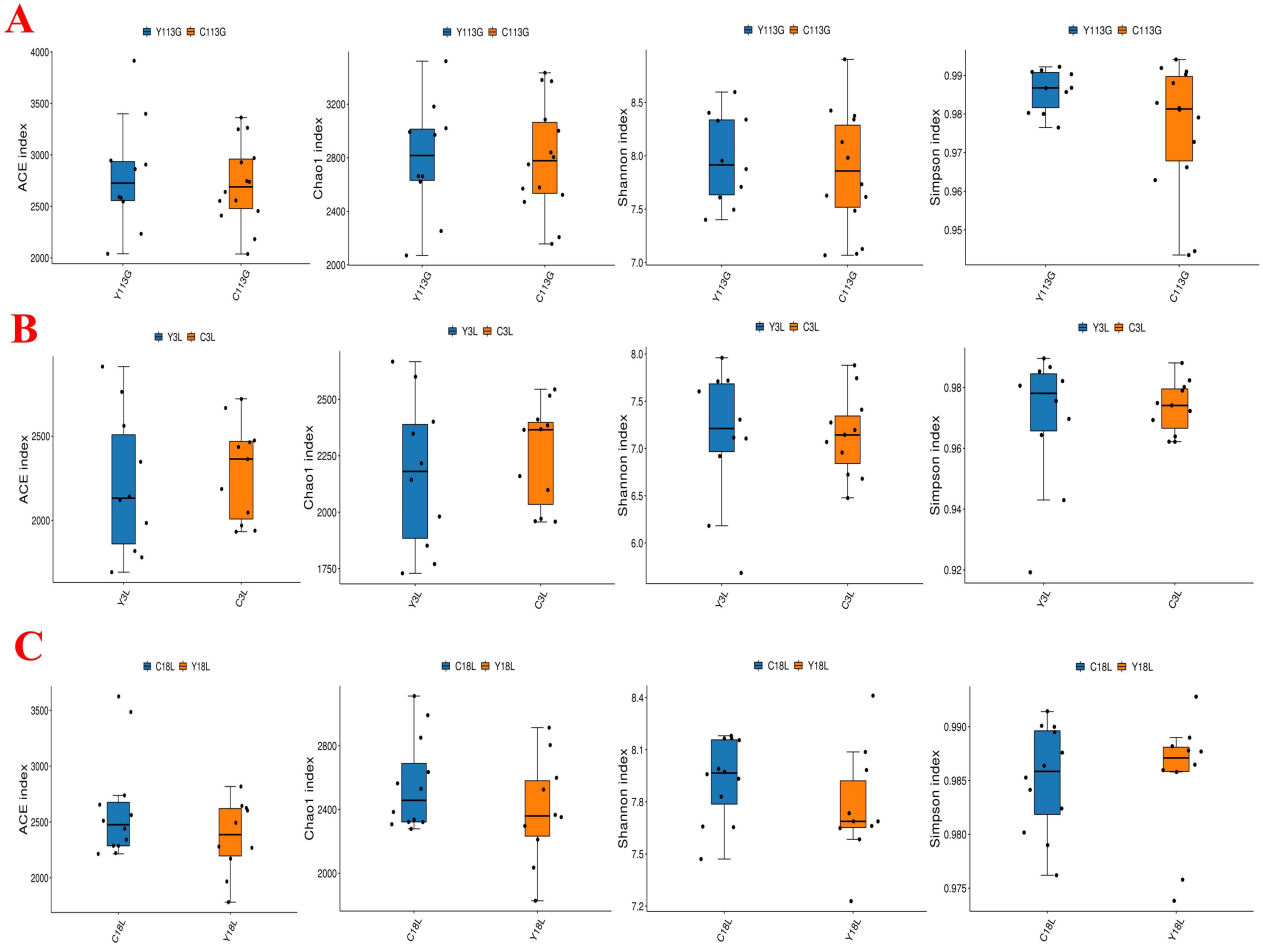
The effect of yeast protein supplementation on the alpha diversity index of fecal microbiota in sows at different stages. **(A)** 113G, day 113 of gestation. **(B)** 3L, day 3 of lactation. **(C)** 18L, day 18 of lactation. C, sows fed with control diet; Y, sows fed with yeast protein-supplemented diet.

**Figure 6:**
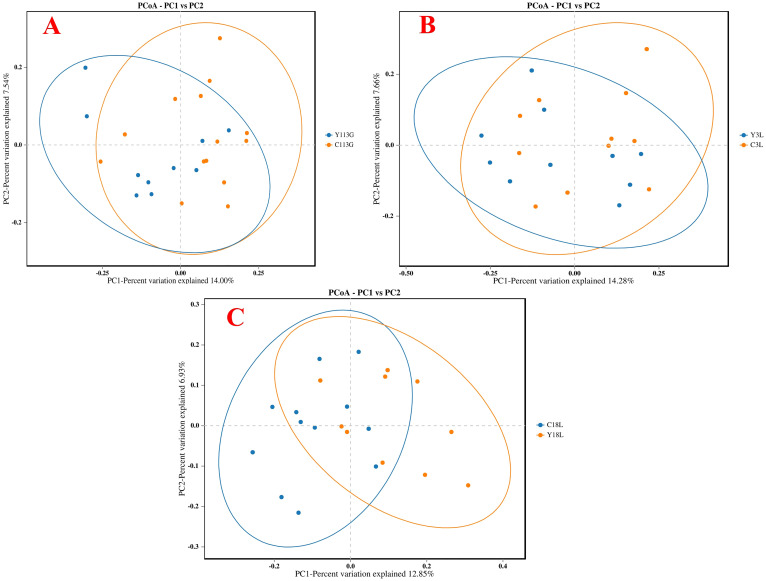
The Principal Coordinate Analysis (PCoA) of bacterial communities in sows fed either a control diet or a yeast protein-supplemented diet. **(A)** 113G, day 113 of gestation. **(B)** 3L, day 3 of lactation. **(C)** 18L, day 18 of lactation. C, sows fed with control diet; Y, sows fed with yeast protein-supplemented diet.

The alpha diversity analysis revealed no significant differences in the ACE index, Chao1 index, Shannon index, and Simpson index between control and yeast protein-supplemented sows on day 113 of gestation, day 3 of lactation, and day 18 of lactation (**Figures 5A–C**; *P* > 0.05). Beta-diversity assessment through PCoA based on the Bray-Curtis distance matrices demonstrated no clear clustering between the two groups at any sampling timepoint (*P* > 0.05; **Figures 6A–C**).

**Figure 7:**
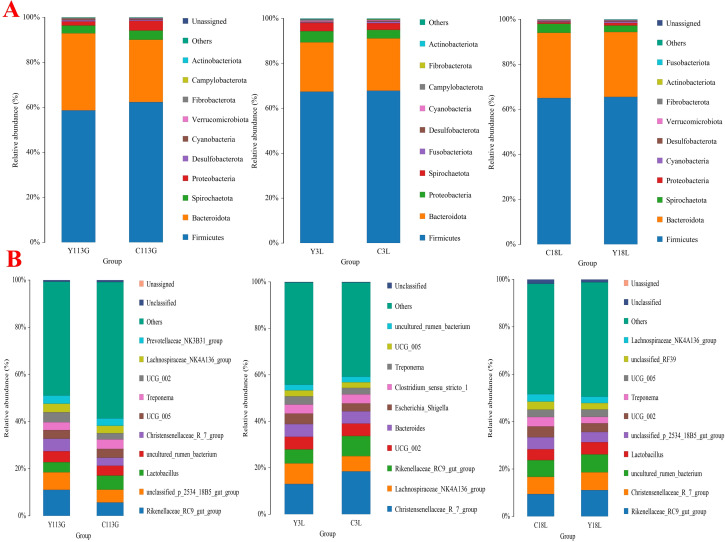
Relative abundances of top 10 bacteria at levels of phyla **(A)** and genera **(B)** in sows fed either a control diet or a yeast protein-supplemented diet at different stages. C, sows fed with control diet; Y, sows fed with yeast protein-supplemented diet.

The community composition at the phylum level in both control sows and yeast protein-supplemented sows demonstrated Firmicutes and Bacteroidetes as the dominant phyla across all samples (**Figure 7A**), consistent with previous observations in different sow hybrids. The bacterial community composition of the top 10 genera during late gestation or lactation is displayed in **Figure 7B**, which shows the same pattern as previously observed in different sow hybrids.

**Figure 8:**
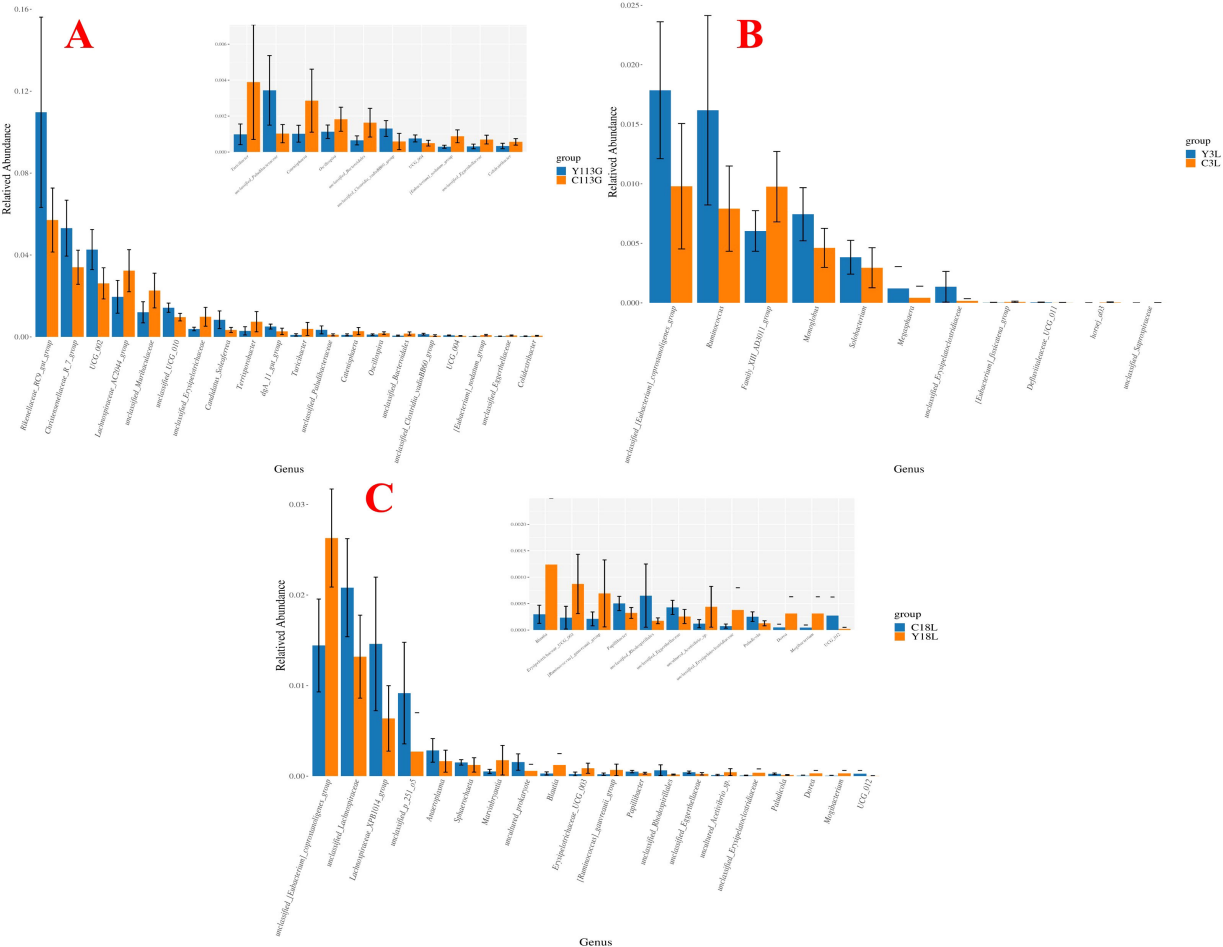
The effect of yeast protein supplementation on the differential bacterial genera in fecal microbiota of sows at different stages. **(A)** 113G, day 113 of gestation. **(B)** 3L, day 3 of lactation. **(C)** 18L, day 18 of lactation. C, sows fed with control diet; Y, sows fed with yeast protein-supplemented diet.

Wilcoxon rank-sum test for the differential microbial genera in the feces of control sows and yeast protein-supplemented sows are shown in **Figure 8**. On day 113 of gestation, yeast protein supplementation exhibited elevated relative abundances of *Rikenellaceae_RC9_gut_group*, *Christensenellaceae_R_7_group*, and *UCG_002* compared to control sows (*P* < 0.05; **Figure 8A**). On day 3 of lactation, yeast protein supplementation demonstrated enriched relative abundances of *Ruminococcus* and *unclassified_[Eubacterium]_coprostanoligenes_group*, but reduced *Family_XIII_AD3011_group* compared to LY sows (*P* < 0.05; **Figure 8B**). On day 18 of lactation, yeast protein supplementation significantly increased the relative abundance of *unclassified_[Eubacterium]_coprostanoligenes_group*, but decreased the abundances of *unclassified_Lachnospiraceae* and *Lachnospiraceae_XPB1014_group* compared to LY sows (*P* < 0.05; **Figure 8C**).

## 4 Discussion

The present study demonstrated no significant differences in most reproductive and lactation parameters between LY and LLY sows, consistent with earlier research by Sun et al. (2020) and Wang et al. (2021). Furthermore, our study revealed replacement of fishmeal with yeast protein (at a 2.6% inclusion rate) during late gestation to weaning tended to reduce the mummification rate of fetuses, although no statistically significant effects were observed on other reproductive and lactation performance parameters. While previous studies have highlighted the beneficial effects of yeast products as functional additives in improving sow performance, the impact of yeast protein substitution for fishmeal on sow productivity remains elusive (Chen et al., 2024). Our findings align with previous studies reporting comparable reproductive and lactation performance in sows supplemented with either live yeast (Le Floc′h et al., 2022; Xia et al., 2022) or yeast-derived products (Chance et al., 2022) at low-dose ranges of 0.01% to 0.125%. This consistency extends to recent research by Chen et al. (2024), who observed similar outcomes when replacing fishmeal with 0.5% to 2.0% yeast protein supplementation from day 103 of gestation to weaning. However, conflicting evidence exists in the literature. Several studies have reported increased numbers of live-born piglets (Hasan et al., 2018; Bass et al., 2019) and enhanced piglet birth weight (Taylor-Pickard et al., 2017) following supplementation with live yeast or yeast derivatives supplementation at doses ranging from 0.08% to 0.2%. Notably, in addition to variations in yeast product types, the timing of supplementation may contribute to these discrepancies. The aforementioned studies demonstrating improved farrowing outcomes implemented supplementation throughout the entire gestation, whereas our intervention commenced during late gestation. This temporal difference suggests that earlier initiation of yeast protein supplementation during gestation might enhance embryonic survival and consequently increase live-born piglets.

In addition, our study indicated that yeast protein supplementation exerted no significant effects on sow lactation performance, contrasting with several previous research. Previous studies have demonstrated increased sow feed intake (Tan et al., 2021; Zhao et al., 2022), elevated milk yield and composition (Peng et al., 2020; Zhao et al., 2022; Chen et al., 2024), and improved pre-weaning piglet growth performance (Zhao et al., 2022; Liu et al., 2023; Chen et al., 2024; Kim and Duarte, 2024) by utilizing various yeast-based products, including live yeast, yeast culture, yeast extract, yeast hydrolysates, and yeast protein. The absence of significant alterations in colostrum composition and milk yield observed in our study may directly explain the unchanged piglet growth performance during lactation. Notably, we identified that yeast protein supplementation significantly attenuated sow backfat loss during lactation. Excessive loss of backfat and body mobilization may compromise subsequent reproductive performance through increasing the weaning to estrus interval of sows (Thaker and Bilkei, 2005) and reducing ovulation rates and embryonic survivals (Van den Brand et al., 2000; Vinsky et al., 2006). Our results align with previous research documenting the adipose-preserving effects of yeast-derived supplements, including yeast extracts and live yeast (Tan et al., 2021; Sun et al., 2022). As proposed by Sun et al. (2022), the metabolic demands of parturition drive substantial energy expenditure that precipitates backfat depletion. The supplementation of yeast fermentation or culture products, characterized by high nutrient density and bioavailability, appears to enhance the energy reserves of sows, effectively counterbalancing this catabolic process with no effect on feed intake as evidenced in our results.

Serum immunoglobulin concentrations serve as critical indicator of humoral immune response in animals. Changes in these protein levels have been demonstrated to affect animal productivity and immunity. IgA, IgG, and IgM are the main immunoglobulins in the body fluids of humans and animals. IgA governs mucosal immunity within the gastrointestinal tract, IgG is the most important immune factor in secondary immune responses, and IgM is the main immune factor involved in primary immune responses (Hăbeanu et al., 2022). Our study revealed a statistically significant reduction in serum IgM concentrations in LLY sows compared to LY sows on day 18 of lactation. Given the pivotal role of IgM in innate immunity, particularly its ability to neutralize pathogens and act as a cell and pathogen signaler for lysis by complementary cells (Keyt et al., 2020), the observed deficiency suggests diminished immunocompetence in LLY sows during late lactation. Additionally, we found that yeast protein supplementation reduced IgG concentration in both serum on day 18 of lactation and colostrum. This result contrasts with previous studies utilizing yeast-derived product supplementation, which reported either no alterations in colostrum immunoglobulin profiles (Hasan et al., 2018; Bass et al., 2019; Le Floc′h et al., 2022; Xu et al., 2023) or elevated immunoglobulin levels in blood (Xia et al., 2022; Zhao et al., 2022) or colostrum (Quinn et al., 2001; Jang et al., 2013; Zanello et al., 2013; dos Santos et al., 2023). The observed discrepancies may stem from the type of yeast-derived products and the length and rate of inclusion. The yeast protein used in our study, derived from dried yeast through polysaccharide-depletion enrichment, primarily consists of yeast cell proteins and metabolic products. In contrast, yeast products in previous studies retained β-glucan-rich cell walls, known to enhance both innate and adaptive immunity (Zhen et al., 2020; Byrne et al., 2021; Bi et al., 2022; Rhayat et al., 2023). Notably, yeast protein supplementation reduced serum IL-1β concentration by 45.6% in sows on day 18 of lactation, a key pro-inflammatory cytokine linked to systemic inflammation (Parrilla et al., 2020). This anti-inflammatory effect aligns with findings by Fu et al. (2023) and Fan et al. (2024), who observed that supplementation with yeast hydrolysate or yeast fermentation products reduced serum IL-1β concentrations in weaned piglets challenged with lipopolysaccharide or *Salmonella* typhimurium.

The gut microbiota plays a critical role in animal health by regulating key physiological functions, including nutrient metabolism, growth and development, intestinal barrier maintenance, immune modulation, and protection against pathogen invasion (Barathan et al., 2024). On day 18 of lactation, the relative abundance of *dgA_11_gut_group and Limosilactobacillus* was significantly lower in LLY sows. *DgA-11_gut_group* is involved in metabolism of amino acids, energy, and lipids (Sun et al., 2019). A recent study by Li et al. (2024) demonstrated that supplementation with mulberry 1-deoxynijirimycin increased the relative abundance of *dgA-11_gut_group*, thereby reducing inflammatory responses in rabbits. Chang et al. (2024) demonstrated that the relative abundance of *Limosilactobacillus* positively correlated with the serum IL-6 concentrations in sows. And this genus has been shown to possess antimicrobial properties and intestinal immune functions, mitigating inflammation and colitis through NF-kB signaling pathway regulation (Liu et al., 2022). Thus, the reduced *dgA-11_gut_group* and *Limosilactobacillus* abundance in the feces of LLY sows may explain their lower serum IgM concentrations compared to LY sows observed on day 18 of lactation in this study.

Our study demonstrated that yeast protein supplementation did not alter the α-diversity or β-diversity of gut microbiota in sows, indicating no effects on microbial species diversity and richness in either LLY or LY sows. These findings align with reports by Hasan et al. (2018) and Zhao et al. (2022), though contradictory results exist. Notably, Ma et al. (2023) observed significant increases in Shannon, Simpson, and Sobs indices following gestational yeast culture supplementation. We propose these discrepancies may stem from variations in yeast product types and the timing of supplementation. Our supplementation spanned late gestation to weaning, while Ma’s study lasted from day 30 of gestation to weaning. Our results revealed yeast protein supplementation significantly increased relative abundance of *Rikenellaceae_RC9_gut_group*, *Christensenellaceae_R_7_group*, and *UCG_002* in sows on day 113 of gestation. These findings align with previous research by Ma et al. (2023), who reported that yeast culture supplementation effectively increased intestinal abundances of *Rikenellaceae_RC9_gut_group* and *Prevotellaceae_NK3B31_group* in lactating sows. Cai et al. (2021) reported significantly reduced *Rikenellaceae_RC9_gut_group* abundance in mice with intestinal inflammation and malnutrition. *Christensenellaceae_R_7_group* has been identified as a potential beneficial bacterium contributing to gut homeostasis and immune regulation (Kong et al., 2016). These microbial shifts suggest yeast protein benefits late-gestation gut health in sows. During lactation, yeast protein supplementation increased *Ruminococcus* and *unclassified_[Eubacterium]coprostanoligenes_group* abundances in sows on day 3, with sustained elevation of the latter through day 18. Concurrently, it reduced *Family_XIII_AD3011_group* abundance on day 3 and decreased *unclassified_Lachnospiraceae* and *Lachnospiraceae_XPB1014_group* on day 18. These findings align with previous studies. Ma et al. (2023) documented similar reductions in *Lachnospiraceae_XPB1014_group* and *Terrisporobacter* in yeast culture-supplemented sows, while Zhao et al. (2022) observed increased *Ruminococcus* and decreased *Bacteroidales* abundances with yeast culture supplementation. The *Ruminococcus* genus, comprising two fiber-degrading species, hydrolyzes plant fibers into cellulose and hemicellulose, which are subsequently fermented to volatile fatty acids (VFAs) (Jami and Mizrahi, 2012). This suggests yeast protein-induced increases in *Ruminococcus* abundance may affect cellulose digestion, VFA production, and energy utilization efficiency, as observed in Tibetan sheep (*Ovis aries*) supplemented with selenium yeast (Cui et al., 2021). Bai et al. (2024) demonstrated that *Eubacterium coprostanoligenes* could stimulate mucin production in goblet cells, enhancing intestinal mucus barrier integrity to prevent microbial invasion and reduce the inflammatory response. Notably, *Family_XIII_AD3011_group* shows negative correlations with acetic acid concentrations (Shi et al., 2020) and impaired disease resistance in Tibetan pigs (Shang et al., 2022). As a conditional pathogen, this genus may induce hypoglycemia through dysbiosis-driven insulin hypersecretion and has been associated with human depression and metabolic disorders (Zhang et al., 2017). In growing pigs, *Lachnospiraceae_XPB1014*_*group* abundance negatively correlates with body fat weight (Hu et al., 2019), potentially explaining our observed 30.5% reduction in lactational backfat loss in sows with yeast supplementation through its suppression. Collectively, these microbial shifts likely account for the 45.6% reduction in serum IL-1β concentrations observed in yeast-supplemented sows on day 18 of lactation, indicating yeast protein promotes beneficial bacterial proliferation, and suppresses pathogenic proliferation, thereby improving gut health in lactating sows.

## 5 Conclusion

LY and LLY sows exhibited comparable reproductive performance, immune function, and gut microbiota, demonstrating the practical feasibility of retaining LLY sows for commercial breeding. Yeast protein supplementation as a substitute for fishmeal during late gestation and lactation significantly reduced lactational backfat loss and moderately attenuated inflammatory response. This effect was likely mediated through selective gut microbiota modulation by promoting beneficial genera such as *Christensenellaceae_R_7_group*, *Ruminococcus* and *Eubacterium coprostanoligenes*, while suppressing specific genera including *Family_XIII_AD3011_group* and *Lachnospiraceae_XPB1014*_*group*. These findings indicate that yeast protein substitution for conventional high-protein ingredients not only reduces feed costs but also improves reproductive performance, immune function, and gut microbiome homeostasis in sows.

## Data Availability

The datasets presented in this study can be found in online repositories. The names of the repository/repositories and accession number(s) can be found below: https://www.ncbi.nlm.nih.gov/, PRJNA1245321.
